# Case Report: Urinary tract isolation of *Cronobacter sakazakii* in an oncohaematological patient in Southern Italy

**DOI:** 10.3389/fonc.2026.1736054

**Published:** 2026-01-30

**Authors:** Antonella Mecca, Debora Carrante, Giovanna Rosaria Mansueto, Gabriella Bianchino, Fabiana Crispo, Biagina Campisi

**Affiliations:** 1 Unit of Clinical Pathology IRCCS-CROB Referral Cancer Center of Basilicata, Rionero in Vulture, PZ, Italy; 2 Hematology and Stem Cell Transplantation Unit, IRCCS-CROB Referral Cancer Centre of Basilicata, Rionero in Vulture, PZ, Italy; 3 Laboratory of Preclinical and Translational Research, IRCCS-CROB Referral Cancer Center of Basilicata, Rionero in Vulture, PZ, Italy

**Keywords:** adult oncohaematological patient, *Cronobacter sakazakii*, emerging pathogen, opportunistic infection, urinary tract infection

## Abstract

**Background:**

*Cronobacter sakazakii* is an emerging Gram-negative opportunistic pathogen, mostly associated with severe neonatal infections. In adults, infections are rare and usually occur in immuno-compromised or elderly patients. Urinary tract infections caused by *C. sakazakii* in oncological adults are extremely uncommon.

**Case:**

We report a 66-year-old Caucasian male with acute myeloid leukaemia (AML) with a history of bladder carcinoma. The patient was admitted to the Haematology ward with fever (38.5°C), dysuria, pyuria, abdominal pain, and diarrhoea. He was already undergoing treatment at our Institute for AML and had completed the third cycle of liposomal daunorubicin/cytarabine (Vyxeos) 23 days prior to admission. Urine culture revealed *C. sakazakii* infection, confirmed on two different culture media. Blood and stool cultures were negative. The isolate was fully susceptible to all tested antibiotics. Empirical therapy with piperacillin/tazobactam was initiated, leading to rapid resolution of fever and urinary symptoms. Follow-up urine cultures after one week were negative, and the patient was discharged.

**Conclusions:**

This case highlights a rare urinary tract infection caused by *C. sakazakii* in an immunocompromised adult. Conventional culture-based methods, with confirmation on two different media, enabled accurate identification of the pathogen. Prompt empirical antimicrobial therapy resulted in rapid clinical improvement and complete recovery. Reporting such cases contributes to awareness of this emerging pathogen in adult oncological patients and underscores the importance of culture-based diagnosis to guide effective management.

## 1 Introduction

*Cronobacter sakazakii* is a Gram-negative, xerotolerant, facultatively anaerobic, motile, rod-shaped bacterium, generally oxidase-negative and non-spore-forming (1, 2). Formerly classified as *Enterobacter sakazakii* until 2007, it was reclassified into the genus *Cronobacter* (3).

*C. sakazakii* is an emerging pathogen, primarily causing severe neonatal infections such as necrotizing enterocolitis, sepsis, and meningitis, often linked to contaminated powdered infant formula (4, 5). In adults, infection is rare and can result in bacteraemia, osteomyelitis, and opportunistic urinary tract infections (UTIs), particularly in immunocompromised or frail elderly patients (6, 7). Reports describing *C. sakazakii* urinary tract infections in adult patients with haematological malignancies are exceptionally uncommon and available scientific literature provides only sparse documentation, underscoring the rarity of such occurrences.

This case report presents an adult male undergoing treatment for AML at IRCCS-CROB, Rionero in Vulture (PZ), who was diagnosed with a urinary tract infection due to *C. sakazakii*.

## 2 Case presentation

A 66-year-old Caucasian male with AML myelodysplasia-related changes, without molecular rearrangements, and high cytogenetic risk (karyotype: 47,XY, + 8; 46,XY,del(7)), and a history of bladder carcinoma, with a permanent ureterostomy since 2000, presented to our Institute with fever (38.5°C), dysuria, pyuria, abdominal pain, and diarrhoea (**Table 1**). He was admitted to the Haematology ward and underwent blood tests, revealing elevated inflammatory markers: CRP 188 mg/L, ESR 44 mm/h, procalcitonin 9.62 ng/mL, WBC 4270/µL with 82.2% neutrophils (3.5 x 10^^^3/µL neutrophils), and 295 x 10^^^3/µL platelets (**Table 2**). The general symptoms and laboratory findings were consistent with a systemic inflammatory response, likely due to a bacterial infection. Given the immunocompromised status caused by AML and chemotherapy with Vyxeos, the patient was at high risk for infectious complications.

**Table 1:** Clinical timeline summarizing the patient’s diagnosis, chemotherapy, hospital admission, microbiological investigations, antimicrobial therapy, and clinical outcome.

Date	Clinical event
24/04/2025-31/05/2025	Diagnosis of AML with myelodysplasia-related changes; hospitalization.
30/04/2025-09/07/2025	Vyxeos chemotherapy cycles I–III
29/07/2025	Hospital admission; urine and blood cultures; start piperacillin/tazobactam.
30/07/2025	Peripheral blood cultures (second and third samples)
31/07/2025	Stool culture
04/08/2025	Discharge, symptom-free

**Table 2:** Baseline laboratory findings at hospital admission.

Parameter	Value	Reference range
WBC	4270/µL	4000-11000/µL
Neutrophils	82.2%	40%-80%
CRP	188 mg/L	< 10 mg/L
ESR	44 mm/h	<20 mm/hr (Men > 50)
Procalcitonin	9.62 ng/mL	< 0.05 ng/mL
Urine culture	10^6 CFU/mL	—

WBC, white blood cell count; CRP, C-reactive protein; ESR, erythrocyte sedimentation rate; CFU, colony-forming unit.

Urinalysis performed using Sysmex UF 5000 revealed cloudy urine, erythrocytes (15–20/field), numerous degenerate leukocytes, nitrites, bacteria (95000/µL), proteinuria (15 mg/mL), specific gravity 1.011, pH 5.5, and urobilinogen 0.2 mg/mL. Urine culture was performed using a calibrated 10µL sterile loop on ORI Chromogenic Agar (BD Diagnostic Systems), a non-selective medium suitable for direct identification and differentiation of urinary pathogens. Plates were incubated at 37°C for 24 h under aerobic conditions.

**Figure 1:**
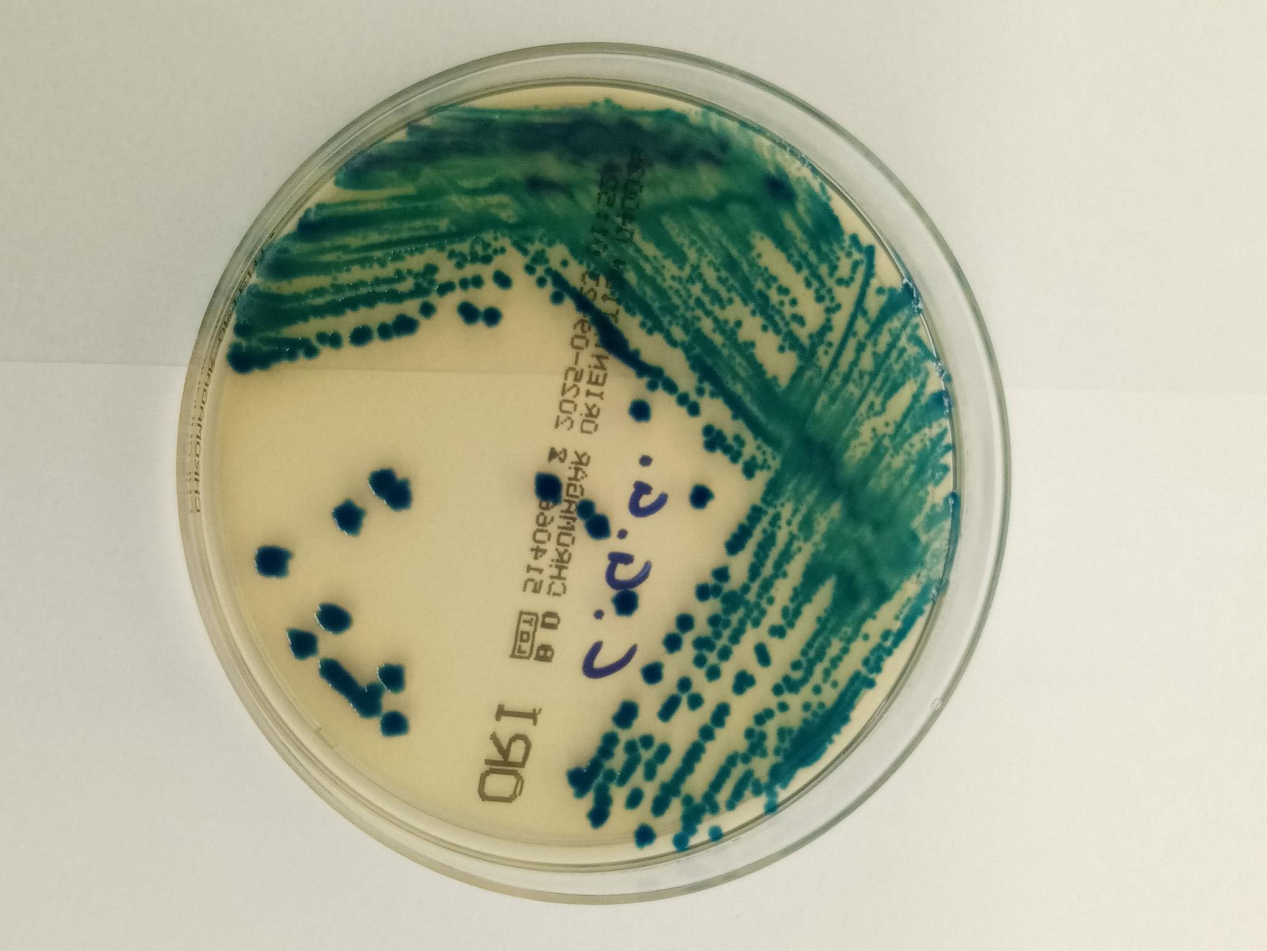
Growth of *Cronobacter sakazakii* on ORI chromogenic agar after 24 h of aerobic incubation at 37°C, showing green–blue creamy colonies with irregular margins.

**Figure 2:**
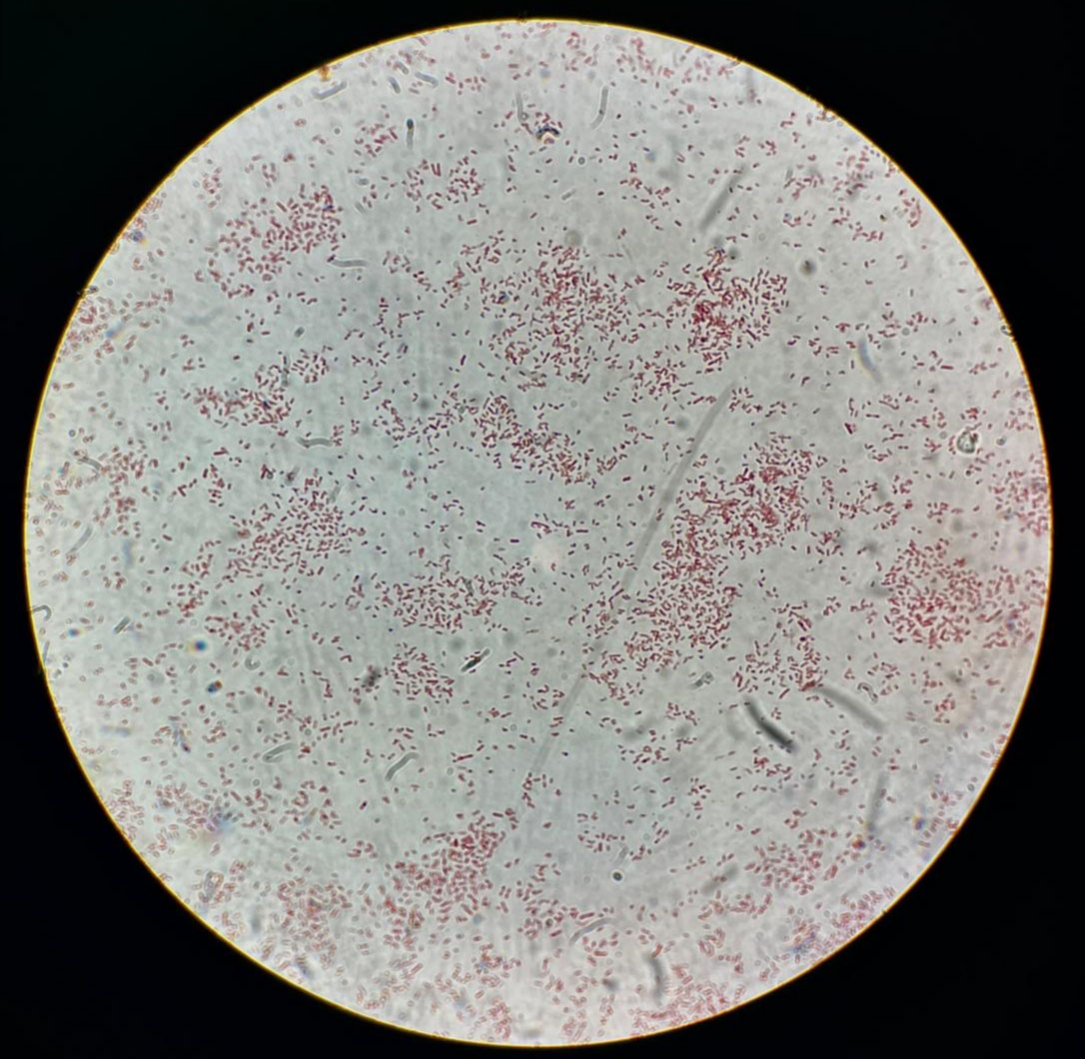
Gram staining of the urinary isolate showing Gram-negative, rod-shaped bacteria. Image acquired at 1000× magnification using oil immersion microscopy on an Olympus BHS microscope. Kindly provided by Dr. Francesca Rocco.

After incubation, significant bacterial growth (≥10^^^6 CFU/mL) of green/blue, creamy colonies with irregular edges was observed (**Figure 1**). Gram staining revealed rod-shaped, red-stained Gram-negative bacteria (**Figure 2**).

**Figure 3:**
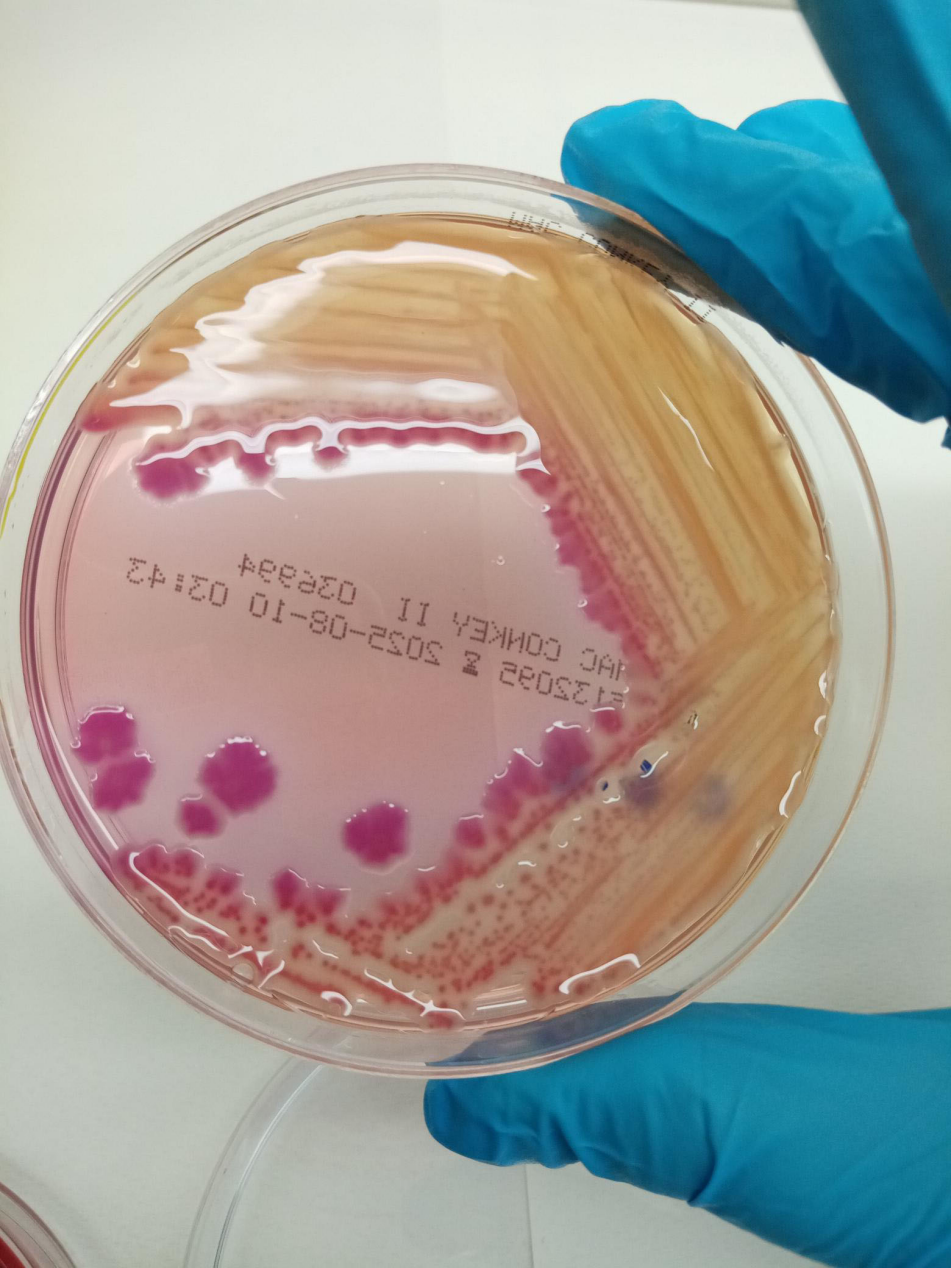
Growth of *Cronobacter sakazakii* on selective MacConkey agar for Gram-negative bacteria after 24 h of aerobic incubation at 37°C.

Identification of the Gram-negative isolate was performed using the Vitek2 system (bioMérieux) with a GN identification card, while antimicrobial susceptibility testing was carried out using the AST-437 card and interpreted according to EUCAST 2024 clinical breakpoints, identifying the bacterium as *Cronobacter sakazakii*. Confirmation was achieved by subculturing on both ORI chromogenic agar and selective MacConkey agar, which corroborated the presence of *C. sakazakii* (**Figure 3**).

Antimicrobial susceptibility testing demonstrated that the isolated microorganism was susceptible to all tested antibiotics (**Table 3**).

**Table 3:** Antimicrobial susceptibility of the *Cronobacter sakazakii* isolate.

Antibiotic	Interpretation	MIC(µg/mL)
Amikacin	S	2
Cephalexin	S	16
Cefixime	S	<=0.25
Cefpodoxime	S	<=0.25
Ceftazidime	S	0.25
Ceftriaxone	S	<=0.25
Ciprofloxacin	S	<=0.06
Ertapenem	S	<=0.12
Gentamicin	S	<=1
Levofloxacin	S	<=0.12
Meropenem	S	<=0.25
Trimetoprim/Sulfamethoxazole	S	<=20
Amoxicillin/clavulanate for UTI	S	<=4

Interpretation based on EUCAST 2024 clinical breakpoints. Legend: S = Susceptible, I = Intermediate, R = Resistant.

Empirical therapy with piperacillin/tazobactam (4.5 g three times daily) was initiated based on the patient’s presenting symptoms while awaiting the results of urine culture and antimicrobial susceptibility testing. Subsequent stool cultures were negative for *Salmonella*, *Shigella*, *Campylobacter*, *Yersinia*, and *Clostridium*, and *C. sakazakii* was not detected, likely due to the ongoing effect of the empirical treatment. Additionally, three peripheral blood cultures and one obtained from the patient’s PICC line all yielded negative results, supporting the conclusion that the infection was confined to the urinary tract and caused by the isolated *C. sakazakii* strain.

The patient’s symptoms resolved rapidly, and for this reason no adjustment to the antimicrobial regimen was necessary. Within 24 hours of initiating antibiotic therapy, the patient became afebrile, and inflammatory markers—particularly procalcitonin—showed progressive improvement from the third day onward. Therefore, continuation of the same antimicrobial treatment was considered appropriate. Follow-up urine cultures were negative after one week. Intravenous therapy was administered for seven days, after which the patient was discharged in stable condition, without further therapies at home.

This report follows the CARE guidelines for clinical case reports.

## 3 Discussion and conclusions

*Cronobacter* species have been isolated from clinical samples (blood, cerebrospinal fluid, stool), food products (dairy products, powdered infant formula, meat, and vegetables), and environmental sources (water and soil) (8–13). Their pathogenic potential is largely related to a remarkable ability to survive in highly desiccated environments, such as powdered milk. As a foodborne pathogen, *C. sakazakii* is frequently detected in dairy products, particularly powdered infant formula (PIF), which represents both the main vehicle and a significant source of contamination (4, 9–12). The European Food Safety Authority (EFSA) has also linked *Cronobacter* contamination to formula production processes and domestic reconstitution practices (11). In neonates, *C. sakazakii* infections can be severe and are associated with high mortality rates (40–80%), especially in preterm infants with immature immune systems (13). In adults, infections are rare and generally less severe. In the literature, urinary tract infections caused by *Cronobacter* species have been reported in patients both with and without comorbidities (6, 14), as well as cases of sepsis, cholangitis, pulmonary infections and pyosalpinx, most often in immunocompromised patients with comorbidities or frail elderly patients (7, 15–17).

The present study is a retrospective single-patient case report describing a rare occurrence of urinary tract infection caused by *C. sakazakii* in an adult oncohaematological patient presenting with dysuria, fever (38.5°C), and abdominal pain. Although the source of infection could not be identified, the patient’s medical history likely contributed to increasing susceptibility to infections. Previous bladder carcinoma treated with cystoprostatectomy and permanent ureterostomy, combined with immunosuppression, due to AML with myelodysplasia-related changes and ongoing chemotherapy, markedly elevated the risk of opportunistic infections, compared to immunocompetent individuals. Importantly no additional cases of *Cronobacter* infection were detected in the Haematology ward or in other hospital departments during the same period. This represents the first documented isolation of *Cronobacter* species in our hospital, further underscoring the rarity of such infections in adult clinical settings.

Moreover, our case reinforces the importance of considering rare pathogens in immunocompromised patients, which are high-risk individuals. Early recognition and accurate microbiological identification are critical to initiating effective therapy and preventing complications. Conventional culture-based diagnostic methods—using two different culture media (ORI chromogenic agar and MacConkey agar)—were sufficient to detect a rare *C. sakazakii* infection in an immunocompromised AML patient. Although genotyping of the *C. sakazakii* strain was not performed, as it is not fundamental for diagnostic purposes, such detailed molecular characterization could provide valuable epidemiological insights and contribute to a more comprehensive understanding of this opportunistic pathogen. Indeed, accurate pathogen identification and antimicrobial susceptibility testing enabled prompt initiation of effective empirical therapy, resulting in rapid clinical improvement. In the current urgent fight against multidrug-resistance in hospitals, the appropriateness of antibiotic use, especially in the presence of susceptible pathogens, represents an essential element to ensure clinical efficacy and to reduce the risk of acquired secondary resistance, safeguarding public health. In this context, ongoing research offers alternative and combination treatments to counteract emerging pathogens, including *C. sakazakii* (18).

In conclusion, by reporting this case of *C. sakazakii* opportunistic infection in an oncohaematological patient, we underscore the extreme rarity of this pathogen as a cause of UTI in patients with AML and highlight the need for vigilance regarding rare opportunistic pathogens when managing infections in immunocompromised patients, especially when conventional pathogens are not identified. To date, no case reports or case series explicitly documenting *C. sakazakii* UTIs in oncohaematological patients have been identified in the mainstream literature (e.g., PubMed-indexed case reports or reviews), although sporadic opportunistic infections caused by *C. sakazakii* have been reported in adults with comorbidities or immunosuppression (6, 7, 13–16). In this vulnerable population, *C. sakazakii* may cause severe infections and clinically significant symptoms, warranting precise microbiological identification and targeted antimicrobial therapy to prevent adverse outcomes.

## Data Availability

The original contributions presented in the study are included in the article. Further inquiries can be directed to the corresponding author.
